# *Tomato yellow leaf curl virus* (TYLCV-IL): a seed-transmissible geminivirus in tomatoes

**DOI:** 10.1038/srep19013

**Published:** 2016-01-08

**Authors:** Eui-Joon Kil, Sunhoo Kim, Ye-Ji Lee, Hee-Seong Byun, Jungho Park, Haneul Seo, Chang-Seok Kim, Jae-Kyoung Shim, Jung-Hwan Lee, Ji-Kwang Kim, Kyeong-Yeoll Lee, Hong-Soo Choi, Sukchan Lee

**Affiliations:** 1 Department of Genetic Engineering, Sungkyunkwan University, Suwon 440–746, Korea; 2 Crop Protection Division, National Academy of Agricultural Science, Rural Development Administration, Wanju 565–851, Korea; 3 Institute of Plant Medicine, Kyungpook National University, Daegu 702–701, Korea; 4 Biological Resources Research Center, Gyeongsangbuk-do Agricultural Research and Extension Services, Andong 760–891, Korea; 5 Research and Development Bureau, Chungcheongnam-do Agricultural Research and Extension Services, Yesan 340–861, Korea

## Abstract

*Tomato yellow leaf curl virus* (TYLCV) is one of the most well-known tomato-infecting begomoviruses and transmitted by *Bemisia tabaci*. Seed transmission has previously been reported for some RNA viruses, but TYLCV has not previously been described as a seed-borne virus. In 2013 and 2014, without whitefly-mediated transmission, TYLCV was detected in young tomato plants germinated from fallen fruits produced from TYLCV-infected tomato plants in the previous cultivation season. In addition, TYLCV-Israel (TYLCV-IL) was also detected in seeds and their seedlings of TYLCV-infected tomato plants that were infected by both viruliferous whitefly-mediated transmission and agro-inoculation. The seed infectivity was 20–100%, respectively, and the average transmission rate to seedlings was also 84.62% and 80.77%, respectively. TYLCV-tolerant tomatoes also produced TYLCV-infected seeds, but the amount of viral genome was less than seen in TYLCV-susceptible tomato plants. When tomato plants germinated from TYLCV-infected seeds, non-viruliferous whiteflies and healthy tomato plants were placed in an insect cage together, TYLCV was detected from whiteflies as well as receiver tomato plants six weeks later. Taken together, TYLCV-IL can be transmitted via seeds, and tomato plants germinated from TYLCV-infected seeds can be an inoculum source of TYLCV. This is the first report about TYLCV seed transmission in tomato.

*Tomato yellow leaf curl virus* (TYLCV) is a tomato (*Solanum lycopersicum*)-infecting plant virus transmitted by whitefly *Bemisia tabaci*^1^,^2^. It belongs to the genus *Begomovirus* of the family *Geminiviridae* and has a single-stranded circular DNA genome of about 2.8 kb encapsidated in a twinned icosahedral virion^3^. TYLCV-infected tomato plants show severe symptoms such as stunting, leaf curling and yellowing, which cause critical production loss in tomato cultivation^4^. In addition to tomato, other cultivated plants including pepper (*Capsicum* species), common bean (*Phaseolus vulgaris*), cucurbit (*Cucumis* species) and eustoma (*Eustoma grandiflora*) have been reported to be TYLCV hosts^3^,^5^,^6^,^7^,^8^. After the first report from the Middle East in 1931, TYLCV has occurred uninterruptedly in tropical and subtropical areas^1^. In Korea, TYLCV has been reported continuously across the country since the first outbreak in 2008^9^,^10^.

The management of TYLCV in tomato is difficult and expensive both in cultivation under a structure and open field production^11^. Many different approaches for controlling TYLCV disease such as removing whiteflies, killing intermediate weeds, and changing cultivation season have been applied to decrease losses due to TYLCV because a single approach is not frequently effective and certain other approaches cannot be used in different agricultural environments and locations^3^,^12^. Therefore a combination of chemical and biological control techniques for integrated pest management should be employed to reduce the population and migration of the whitefly vector, and minimize or eliminate inoculum sources of TYLCV.

The transmission of most plant viruses depends on vectors such as parasitic fungi, root nematodes, mites, and insects^13^,^14^. Some viruses can also be mediated by the sap of virus-infected plants (mechanical transmission), vegetative propagation and grafting^15^. However, these are restricted to horizontal transmission among neighboring plants^16^. On the other hand, seed transmission can play a critical role in survival from season to season and from parent to offspring^16^,^17^. According to previous studies, there are three major methods of vertical transmission of plant viruses via seed contamination^18^. A few stable viruses, such as tobamoviruses, remain only in the seed coat without embryo contamination and then are transmitted to the seedling after germination^19^. Other methods of seed contamination correspond to virus invasion of the embryo, which is usually protected against virus invasion, either from infected maternal tissues or via infected pollen^18^. Approximately 231 plant virus and viroid diseases have been reported to be seed-transmissible^20^. From tomato plants, *Arabis mosaic virus*, *Pepino mosaic virus*, *Tomato apical stunt viroid* (TASVd), *Tomato black ring virus*, *Tomato chlorotic dwarf viroid* (TCDVd), *Tomato mosaic virus*, and *Tomato streak virus* (TSV) were confirmed as transmittable by seeds^21^,^22^,^23^,^24^,^25^,^26^. Among them, TCDVd exhibited 85.5–94.4% transmission though seeds in tomato, and high transmission yields were also identified from TASVd (80%) and TSV (66%)^21^,^24^.

There have been no previous reports addressing the potential of TYLCV to be a seed-borne virus on any host plant. Begomoviruses including TYLCV have been believed to be inoculated by only whitefly-mediated transmission and artificial inoculation with an infectious clone^27^. In some previous papers, *Abutilon mosaic virus* and *Beet curly top virus* affiliated with the family *Geminiviridae* were listed as seed-borne viruses, but they were considered to be erroneous reports with nomenclature problems^20^,^28^. Therefore, geminiviruses were thought to spread only via insect vectors in nature. Recently, however, *Sweet potato leaf curl virus* (SPLCV) belonging to the genus *Begomovirus* was first reported as a seed-transmissible virus in sweet potatoes^29^. More than 70% of the seeds harvested from SPLCV-infected sweet potato plants were infected by SPLCV. The transmission rate of SPLCV from seeds to seedlings was up to 15%. Southern blot hybridization showing SPLCV-specific single- and double-stranded DNAs in seedlings germinated from SPLCV-infected seeds confirmed SPLCV seed transmission. Therefore, the possibility of TYLCV seed transmission has been reinvestigated based on the seed transmission results of SPLCV as another begomovirus in the family *Geminiviridae*.

Seed-transmissible viruses can be an initial source of inoculum for vector-mediated transmission, and viruses can be disseminated over a long range and perpetuated in seeds^30^. Therefore, understanding the possibility of TYLCV seed transmission is important for plant protection against infection and the spread of TYLCV. The careful investigation of TYLCV transmission through seeds can provide answers to unresolved problems about sporadic TYLCV occurrence in cultivation under structures or in bionurseries without outbreak of whiteflies. In this study, TYLCV detection was performed in floral tissues, seeds and their young seedlings harvested from TYLCV-infected plants inoculated by whitefly-mediated infection and agro-inoculation to evaluate the possibility of TYLCV seed transmission in tomato plants. In addition, we also investigated whether seedlings germinated from TYLCV-infected seeds could act as TYLCV reservoirs for whitefly transmission.

## Results

### TYLCV was detected from newly grown tomato plants under no-whitefly conditions

**Figure 1:**
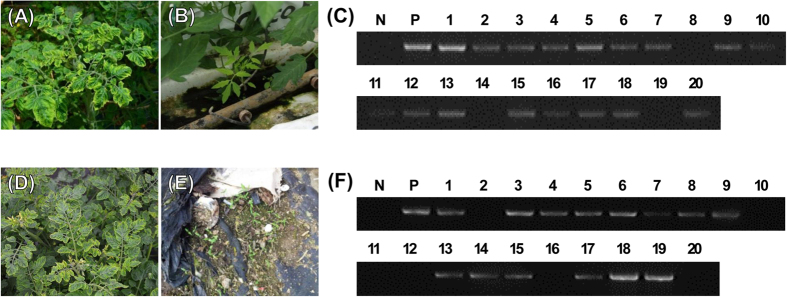
TYLCV detection from seedlings. **(A)** and **(D)** Symptomatic tomato infected by TYLCV. **(B)** and **(E)** Seedlings naturally germinated from fallen fruits of TYLCV-infected tomato plants. **(C)** and **(F)** PCR results using a TYLCV-specific primer set. Lane N, no template control; lane P, positive control with TYLCV-infected tomato genomic DNA; and lanes 1-20, genomic DNA from collected tomato seedlings.

At the tomato-cultivation farm in Jeju Island located in the southern part of Korea, small tomato plants germinated from seeds of fallen tomato fruits from TYLCV-infected tomato plants in the previous cultivation season, were observed and harvested for further TYLCV detection analysis (Fig. 1B). These tomato plants had no visible TYLCV symptoms, but PCR results using the TYLCV-specific primer set showed that 17 plants from 20 randomly selected newly grown small tomato plants were TYLCV infected (Fig. 1C). A similar experiment was performed using tomato plants from just outside of the greenhouse in another tomato farm located in Goheung, Korea, and TYLCV was also detected from 14 of 20 tomato plants (Fig. 1F). No whiteflies were observed on either of these two farms.

### Floral tissues, seeds of TYLCV-infected tomato plants and their seedlings tested positive to TYLCV

**Figure 2:**
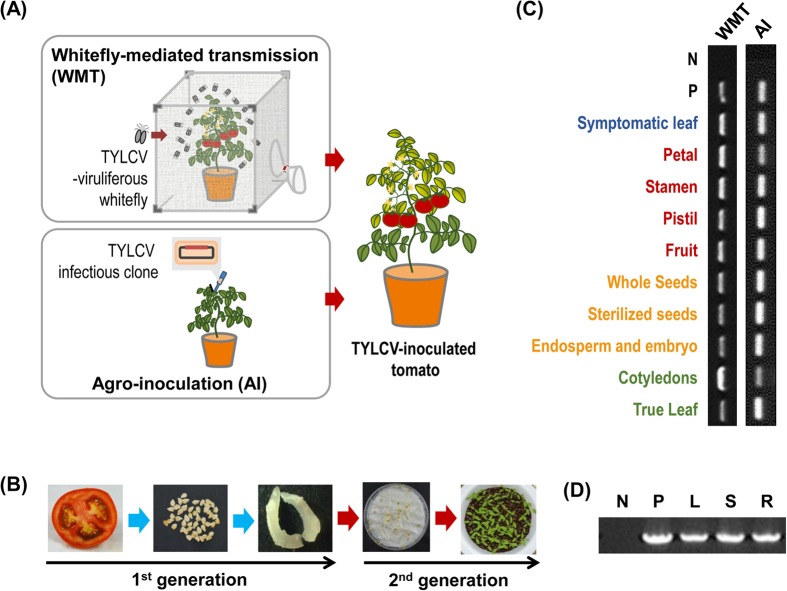
PCR analysis for TYLCV from reproductive tissues and seeds of whitefly-mediated infected and agro-inoculated tomato plants. (**A**) Schematic diagram of preparation of TYLCV-infected tomato plants by whitefly-mediated (WMT) and agro-inoculation (AI) methods. Fruits were harvested from each prepared tomato plant and seeds were collected from tomato fruits. (**B**) Tomato embryos of seeds collected from tomato fruits and cotyledons and true leaves from seeds. (**C**) PCR analysis with symptomatic leaves, floral tissues (petal, stamen and pistil), fruits, whole seeds, sterilized seeds, endosperm and embryos of TYLCV-infected tomato plants and cotyledons and the true leaves of their offspring. Lane N, no template control; and lane P, positive control with TYLCV-infected tomato genomic DNA. (**D**) PCR analysis with leaf, stem and root samples from tomato plants germinated from seeds which were collected from TYLCV-infected tomatoes. Lane N, no template control; lane P, positive control with TYLCV-infected tomato genomic DNA; lane L, leaf; lane S, stem; and lane R, root samples from tomato plants germinated from TYLCV-infected seeds.

All five bulked floral tissues (petal, stamen and pistil respectively) and fruit flesh harvested from TYLCV-infected tomatoes tested positive for TYLCV on PCR analysis. Whole dried seeds, surface-sterilized seeds and embryos with endosperm tissues were also infected with TYLCV (Fig. 2). The TYLCV detection rates of these tissues were determined to be 20–100%, representing the theoretically minimum to maximum values, as described in the Materials and Methods (5/5), respectively (Fig. 2C, Table 1). TYLCV was also detected in all cotyledons (22/26) and true leaves (41/45) of their young tomato plants germinated from seeds, which were collected from whitefly-transmitted TYLCV-infected tomatoes (Fig. 2C, Table 1). In addition, similar results were observed from samples of tomato plants which were agro-inoculated with TYLCV-infectious clones (Fig. 2C, Table 1). All samples, including floral tissues, dried seeds, embryos with endosperms, cotyledons and young seedlings, were infected by TYLCV (Fig. 2C). Analyzed sequences from all tested organs, including floral tissue, seeds and seedlings, exhibited 100% identity with a sequence of the TYLCV-infectious clone (NCBI GenBank accession number JN680149) (data not shown). To analyze the systemic infection of TYLCV in young seedlings, TYLCV was also tested in leaf, stem and root samples of young tomato seedlings, and all samples showed positive TYLCV amplicons in the PCR reaction (Fig. 2D).

### TYLCV DNA accumulated more in seeds and their seedlings harvested from the TYLCV-susceptible tomato cultivar than those of the TYLCV-resistant cultivar after TYLCV infection

**Figure 3:**
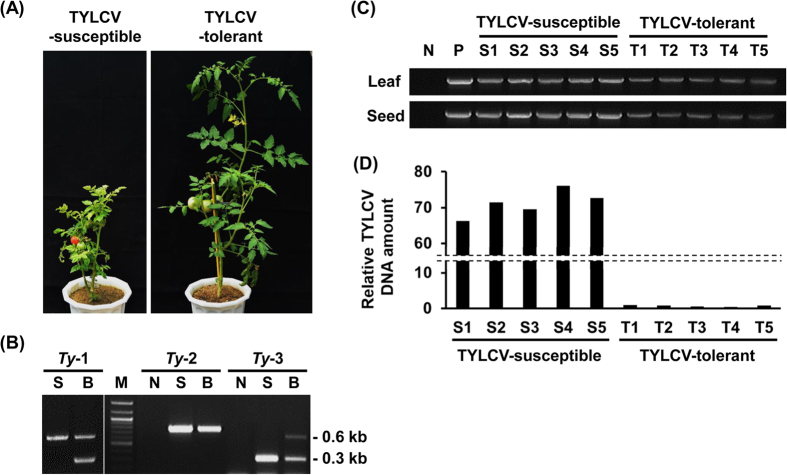
Relationship between different TYLCV DNA amounts and TYLCV susceptibility. **(A)** Symptom development in TYLCV-susceptible Seogwang cultivar tomato showing typical symptoms such as yellowing and curling of leaves and severe stunting (left) and TYLCV-tolerant Bacchus tomato showing no symptoms (right). **(B)** Identification of *Ty*-1, 2, and 3 loci. Two different bands were shown in Bacchus cultivar tomato (left). The *Ty*-2 locus was not confirmed (middle) and the *Ty*-3a/*ty*-3 loci was identified from the Bacchus cultivar tomato (right). Lane M, *iVDye* 100-bp DNA ladder (GenDEPOT, Barker, TX, USA); lane S, TYLCV-susceptible Seogwang cultivar tomato; lane B, TYLCV-tolerant Bacchus cultivar tomato; and lane N, no template control. **(C)** PCR analysis with leaf and seed samples from TYLCV-inoculated TYLCV-susceptible and -tolerant tomato plants. Lane N, no template control; lane P, positive control with TYLCV-infected tomato genomic DNA; lane L, leaf; lane S1-5, genomic DNA form leaves or seeds of TYLCV-susceptible tomato plants; and lane T1-5, genomic DNA from leaves or seeds of TYLCV-tolerant tomato plants. **(D)** Relative DNA amounts of TYLCV in TYLCV-susceptible and -tolerant tomato plants germinated from seeds collected from TYLCV-infected tomatoes. Five individual samples for each cultivar were used for analysis. Relative TYLCV DNA amounts were normalized against T_1_ tomato. Data analyses were conducted using the 2^−ΔΔCt^ method.

To investigate whether or not TYLCV-tolerant tomato plants also transmit TYLCV into seeds and seedlings, TYLCV was agro-inoculated in tomato plants of both a susceptible cultivar (Seogwang cv.) and a resistant cultivar (Bacchus cv.), which were all commercially purchased. TYLCV-tolerant cultivar plants did not show any symptoms, and they grew just like the mock-inoculated healthy tomato plants (Fig. 3A). TYLCV-tolerant cultivar (Bacchus cv.) plants were confirmed as having *Ty*-1 and *Ty*-3a genes by PCR-RFLP analysis; however, TYLCV-susceptible cultivar plants did not have the TYLCV-tolerant gene (Fig. 3B). Figure 3C showed that TYLCV could infect both tomato cultivar plants, and it could also be transmitted to the seeds of two cultivar plants, respectively, even though TYLCV DNA accumulation in TYLCV-tolerant cultivar plants was more reduced than that of TYLCV-susceptible cultivar plants. These different TYLCV DNA accumulation patterns amplified in the seeds of TYLCV-tolerant and TYLCV-susceptible cultivars were similar to the TYLCV DNA accumulation shown in leaf tissues. To analyze whether viral DNA accumulation patterns in seedlings germinated from TYLCV-infected seeds were different between TYLCV-susceptible and -tolerant cultivar plants, a real-time amplification test was conducted with genomic DNA from the seedlings of two different tomato cultivars. Relative amounts of TYLCV DNA in TYLCV-susceptible Seogwang tomato were about 66–164 times more than those in TYLCV-tolerant Bacchus tomato (Fig. 3D). Taken together, all TYLCV DNA accumulation patterns shown in leaves, seeds and seedlings showed strong correlations based on TYLCV susceptibility. The high replication efficiency and high viral DNA accumulation that occur in TYLCV-susceptible plants may provide a greater opportunity for transmission of viral DNA to seeds or seedlings than occurs in TYLCV-resistant plants. These data suggested that the TYLCV tolerance gene may not be involved in the seed transmission process.

### Seed-borne TYLCV was transmitted to healthy tomato plants by whiteflies

**Figure 4:**
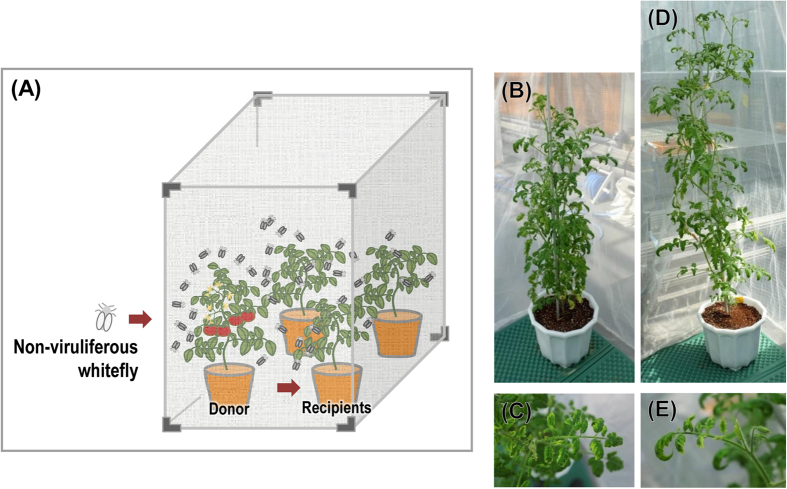
Whitefly-mediated transmission of seed-borne TYLCV from infected to healthy tomato plants. **(A)** Experimental scheme of whitefly-mediated transmission analysis. TYLCV-infected plants germinated from seeds collected from TYLCV-infected tomatoes (donor) and healthy tomato plants (recipients) were put in an insect tent with non-viruliferous whiteflies. During the first 4 weeks, whiteflies became TYLCV viruliferous, and seed-borne TYLCV was transmitted to tomato plants. **(B)** and **(C)** TYLCV-infected donor tomato plants. **(D)** and **(E)** Recipient tomato plants.

**Figure 5:**
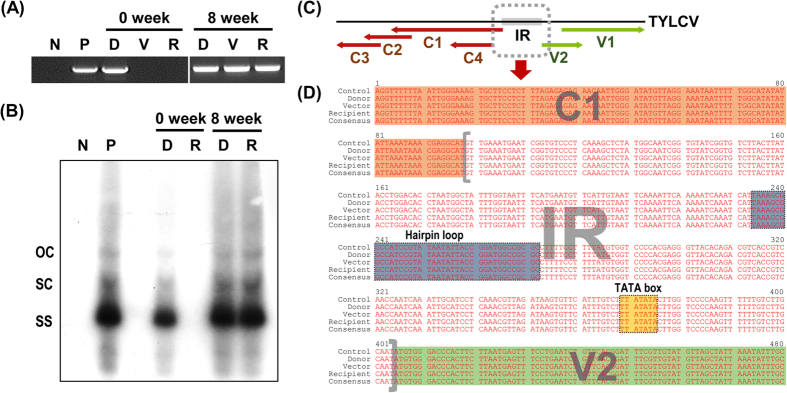
PCR analysis confirming whitefly-mediated transmission of seed-borne TYLCV from infected to healthy tomato plants. **(A)** PCR analysis using a TYLCV-specific primer set and **(B)** Southern hybridization using a TYLCV-specific probe at 0 and 8 weeks after whitefly release in tents. Lane N, negative control; lane P, positive control with TYLCV-infected tomato genomic DNA; lane D, donor plant; lane V, vector (whitefly); and lane R, recipient plant genomic DNA. OC, open-circular double-stranded DNA; SC, supercoiled double-stranded DNA; SS, single-stranded DNA. **(C)** Linearized diagram of TYLCV DNA. Red and green arrows indicate coding sequences and the gray dotted box indicates the region used for sequence analysis containing the intergenic region of TYLCV. **(D)** Multiple sequence alignments of the TYLCV partial genome from donor and recipient plants and a vector with the control sequence (JN680149).

**Figure 6:**
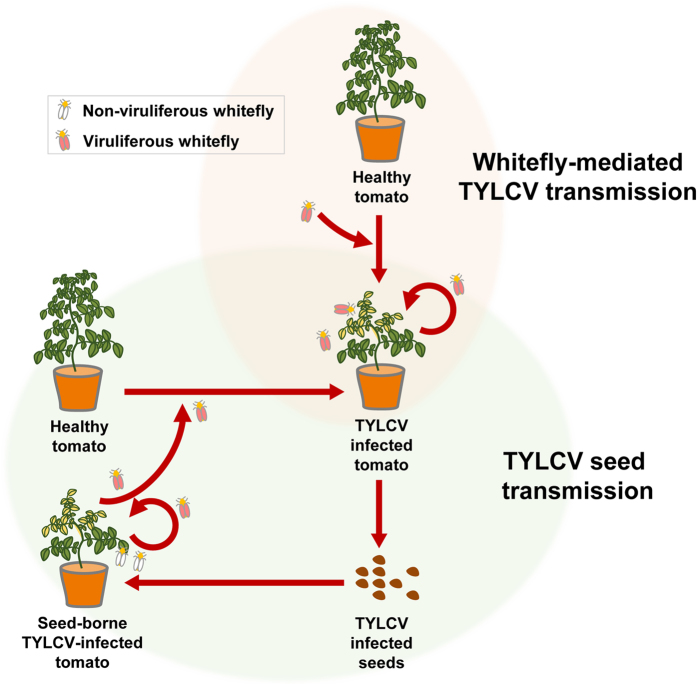
Schematic diagram of the TYLCV disease cycle according to whitefly and seed transmission.

To investigate the possibility of TYLCV transmission from seedlings germinated from TYLCV-infected seeds to new healthy plants via non-viruliferous whiteflies, a transmission assay was performed by placing a donor tomato plant germinated from TYLCV-infected seeds, three healthy receiver tomato plants and around 100 non-viruliferous whiteflies together in an insect-rearing tent (Fig. 4A). After 8 weeks of co-cultivation in an insect-rearing tent, three healthy tomato plants were all infected by TYLCV (3/3) via new viruliferous whiteflies, which obtained TYLCV from a TYLCV-infected donor plant. Three receiver plants produced typical TYLCV disease symptoms on newly developed leaves (Fig. 4E), and these symptoms were confirmed by both PCR and Southern blot hybridization (Fig. 5A,B). In both donor and receiver plants 8 weeks after co-cultivation, Southern blot showed that TYLCV replicated viral DNA in both plants by showing TYLCV-specific ssDNA and dsDNA (Fig. 5B). In the case of new viruliferous whitefly, PCR and Southern hybridization data supported that non-viruliferous whiteflies were TYLCV-viruliferous (Fig. 5A,B). Sequence analysis also supported that all examined samples of the donor plant, receiver plants and whiteflies were infected by the same TYLCV originating from donor tomato plants via whiteflies (Fig. 5C). Taken together, non-virulferous whiteflies transmitted TYLCV from a donor tomato plant, which were germinated from seeds harvested from TYLCV-infected plants, to healthy receiver tomato plants (Fig. 6).

## Discussion

Tomato is one of the most valuable vegetable crops globally, cultivated in many countries^31^. In Korea, tomato has been widely cultivated in almost every area, and consumption of tomato has gradually been increased. However, tomato-infecting pathogens were continuously flowing into Korea and numerous economic losses have been reported^9^,^32^. Among them, TYLCV did a great deal of damage to tomato cultivation, and has been reported consistently throughout the country after the first occurrence in 2008^9^,^10^. Based on sequence analyses, Korean TYLCV isolates were suggested to originate from two Japanese TYLCV isolates^9^. As a hypothesis introduced in the previous report, TYLCV-infected seedlings or viruliferous whitefly can serve as an initial inoculum of TYLCV in Korea, where TYLCV was spread rapidly by non-viruliferous whiteflies observed in many greenhouses since 2005^9^,^20^. In the occurrence or outbreak of some viruses, seeds can be considered the initial viral source (Sastry, 2013). However, in the case of TYLCV as a member of the genus *Begomovirus* and family *Geminiviridae*, the possibility of TYLCV inflow or transmission through seeds was completely excluded because TYLCV was known to be transmitted not by seeds or mechanical inoculation, but only by the insect vector *Bemisia tabaci*^27^,^28^.

Even though TYLCV transmission in nature has been thought to be restricted to whitefly-mediated methods, farmers in Korea periodically raised concerns regarding the potential for seed transmission of TYLCV. By chance, in this study, TYLCV-infected tomato seedlings were identified from whitefly-free conditions in two geographically different regions, Jeju, Jeju Island and Goheung, Chunnam Province (Fig. 1). With the previous documentation of geminivirus seed transmission of SPLCV in sweet potato plants^29^, we were concerned about the vertical transmission of TYLCV from infected tomato plants to offspring via seeds. Like TYLCV, SPLCV, which belongs to the *Begomovirus* genus and *Geminiviridae* family, is the first geminivirus documented to be a seed-transmissible virus. To investigate whether TYLCV could infect floral tissues and seeds from vegetative tissues, floral tissues (petal, stamens and pistils) and seeds (dried seeds, surface-sterilized seeds and embryos) harvested from whitefly transmitted TYLCV-infected tomato plants were tested. In addition, cotyledons and true leaves of young plants were also analyzed. In all tissues, TYLCV was detected with high infection rates (73–91%) (Fig. 2C, Table 1). Sequence analysis of all PCR products showed that TYLCV sequences were identical among the samples, and this result indicated that TYLCV of all samples originated from the initial inoculum (data not shown). Systemic infection of TYLCV in young plants germinated from TYLCV-infected seeds was also confirmed from organ-specific PCR results (Fig. 2D). This result indicated that seed-transmitted TYLCV can infect whole plants, replicate viral DNA, and move systemically just like TYLCV-inoculated via agrobacteria or whiteflies. Seed transmission rates vary from 0% to 100% depending on interactions between the virus and host plant^33^. Seed transmission rates could also vary according to the detection methods used for virus detection such as serological methods or molecular biological methods. New detection methods such as loop-mediated isothermal amplification can yield higher sensitivity and specificity^34^. High seed transmission rates were not a universal feature of plant viruses, but some tomato-infecting viruses and viroids reached high infection rates such as TCDVd (85.5–94.4%), TASVd (80%), and TSV (66%)^21^,^24^. Whitefly-transmitted TYLCV-infected tomato plants showed 20–100% floral infection rates and 20–100% seed transmission rates. Like whitefly-transmitted TYLCV-infected tomato plants, agro-inoculated TYLCV-infected tomato plants also showed similarly high infection rates on different floral tissues (20–100%) and seeds (20–100%) (Fig. 2C, Table 1). Therefore, these inoculation studies indicated that TYLCV seed transmission on tomato plants was confirmed with both inoculation systems, whitefly-mediated infection and agro-inoculation.

The results of different viral genome accumulation rates in young plants germinated from TYLCV-infected seeds between TYLCV-susceptible Seogwang and TYLCV-tolerant Bacchus tomatoes were also confirmed by PCR analysis as well as Southern hybridization (Fig. 3C,D). These TYLCV DNA accumulation patterns in seeds and their seedlings are similar to those in TYLCV-infected tomatoes of these two cultivars (Fig. 3A). Previous studies showed that symptom severity by TYLCV infection was correlated to viral genome methylation, which represented one of the important anti-viral mechanisms of plants called transcriptional gene silencing^35^,^36^. Low TYLCV DNA amounts shown in TYLCV-tolerant tomato are thought to be the result of siRNA production and DNA methylation targeted to viral sequences^35^. Moreover, Fig. 3 showed that TYLCV of a TYLCV-tolerant tomato plant was transmitted via the seeds to seedlings even though viral DNA accumulation was much lower than a TYLCV-susceptible tomato plant. This result may indicate that DNA methylation by the *Ty* gene(s) in a TYLCV-tolerant plant is not a main factor for TYLCV seed transmission because TYLCV of a TYLCV-tolerant plant was able to be transmitted to the seedlings via the seeds. However, in this study, we have not investigated the correlation between the degree of TYLCV DNA methylation in the DNA of the seeds or their seedlings and seed transmission; however, this should be investigated in the near future to understand the different amounts of TYLCV in the seeds of TYLCV-tolerant and TYLCV-susceptible cultivars.

Finally, the transmission of seed-borne TYLCV to other healthy tomato plants by whitefly was tested (Figs 4 and 5) in order to investigate whether TYLCV-infected seeds or their seedlings can be a virus reservoir for further virus spread. Even though TYLCV was identified as a seed-transmissible virus in tomato plants, if TYLCV cannot be transmitted from donor tomato plants germinated from TYLCV-infected seeds to healthy receiver tomato plants, the TYLCV-infected tomato plants may be a dead-end host. Figures 4 and 5 show that TYLCV can be transmitted from a donor plant to a receiver plant via whitefly. Southern data supported that TYLCV in donor and receiver plants can replicate its DNA and move systemically. In addition, in terms of insect transmission, TYLCV in seedlings can be normally acquired by whiteflies and delivered to new healthy plants. This means that TYLCV is a seed-transmissible geminivirus.

TYLCV has caused a great deal of economic damage around the world such that many TYLCV management protocols have been developed^3^,^12^. To prevent TYLCV spread or management, the following approach is recommended: (1) virus- and whitefly-free transplants should be planted, (2) insecticides or insect repellents for whiteflies should be used to reduce whitefly feeding and virus transmission, (3) TYLCV-infected and TYLCV-infected-looking tomatoes should be eliminated from fields and placed in plastic bags immediately at the beginning of the season, (4) plantings of tomatoes should be separated according to time and space from plantings of other TYLCV crop hosts or weeds that are good sources of whiteflies, and (5) TYLCV-resistant tomato cultivars should be used if available in a given production area^12^,^37^. In previous reports and plant pathology fact sheets, the warning about the possibility of TYLCV seed transmission has not been introduced. If TYLCV is a seed-transmissible virus, the summarized TYLCV management protocol is not good enough to prevent TYLCV outbreak in fields. Based on our results, we suggest that two more TYLCV management protocols should be added in succession to the previous protocol: (6) the possibility of TYLCV seed infection should be tested before display on the market, and (7) more strict investigation and drastic monitoring of TYLCV infection or whitefly occurrence on tomato seed gathering fields should be performed.

This is the first report of TYLCV seed transmission in tomato plants. TYLCV can be transmitted by two different transmission cycles: (1) whitefly-mediated transmission and (2) seed-mediated transmission (Fig. 6). Since TYLCV is a notorious virus in tomato plants, in many countries, enormous efforts have been carried out to prevent the infection or spread of TYLCV concerning transmission by whitefly. However, this study showed that TYLCV-infected seeds can be a more critical source for TYLCV spread than TYLCV-viruliferous whiteflies within a country and between countries. Therefore, due to this new observation of TYLCV seed transmission, a new strategy for TYLCV control is needed.

## Methods

### Collecting naturally germinated seedlings from TYLCV-infected tomato fruits

In the spring of 2013 and 2014, tomato seedlings from tomato-cultivating farms on Jeju and Goheung in the Republic of Korea were observed (Fig. 1), and these seedlings were evaluated for TYLCV infection. These small seedlings were not artificially planted, but germinated from the seeds of fallen tomato fruits where TYLCV was present during the last cultivation season. No whitefly was detected in or near the greenhouses, and no egg or larva was present on the abaxial side of leaves. No disease symptoms were identified among the newly planted tomato plants. To prevent the occurrence of TYLCV, cultivated tomato plants and weeds were eliminated and insecticide was sprayed by farmers. For the analysis of the inoculation status, 20 tomato seedlings were randomly gathered from Jeju, and 20 seedlings were collected from Goheung.

### DNA extraction and PCR analysis for TYLCV detection from seedlings

Viral DNA was isolated from the leaves of each tomato seedling using the Viral Gene-spin^TM^ Viral DNA/RNA Extraction Kit (iNtRON Biotechnology, Seongnam, Korea) following the manufacturer’s instructions. PCR was performed using a T100^TM^ Thermal Cycler (Bio-Rad, Hercules, CA, USA) with a final reaction volume of 20 μl containing 20 ng of isolated DNA, 1× *AccuPower*^®^ PCR Master Mix (Bioneer, Daejeon, Korea), and the TYLCV-specific primer set (Table 2). The PCR conditions were as follows: an initial denaturation at 94 °C for 3 min followed by 35 cycles (denaturation at 94 °C for 30 s, annealing at 70 °C for 30 s, and an extension at 72 °C for 30 s), and a final extension at 72 °C for 10 min. Amplified DNA fragments were analyzed and electrophoresed on 1% agarose gels. Each reaction was performed three times.

### Sample collection, DNA isolation and PCR analysis using whitefly- and agrobacterium-mediated inoculated tomato samples

Tomatoes cvs. Seogwang (TYLCV susceptible) and Bacchus (TYLCV tolerant) (Seminis, Anseong, Korea) were planted in a walk-in growth chamber in Sungkyunkwan University, Korea, and fifteen 4-week-old plants were inoculated with agrobacteria containing TYLCV-Isreal (TYLCV-IL)-infectious clones prepared in a previous study^10^, or placed with TYLCV-IL-viruliferous whitefly (*B. tabaci,* Q biotype) in an insect-free BugDorm-2120 insect rearing tent (MegaView Science Education Services Co., Taipei, Taiwan) for five days (Fig. 2A). Before planting tomato plants on soil, seeds were tested for TYLCV contamination or infection in advance. They were transplanted in a greenhouse covered with tightly woven mesh located in Yesan and Iksan to prevent the effects of insects on the plants. Whitefly-mediated TYLCV-inoculated tomato plants were treated with insecticide three times to ensure whitefly removal before transplanting. Four weeks after agrobacterium- and whitefly-mediated inoculation, the leaves, flowers and fruits were collected from symptomatic tomato plants. Seeds were harvested from ripe tomato fruits and washed with distilled water several times to remove the tomato fruit flesh. Washed seeds were surface-sterilized with 70% ethanol for 10 min, and then 10% Clorox for 20 min^38^. Embryos were separated carefully from the seed coat of each seed using a sterile scalpel^30^ (Fig. 2B). The petal, stamen and pistil from the floral tissues were also prepared from mature flowers. Seeds harvested from TYLCV-infected tomato fruits were planted in pots and placed in a BugDorm-2120 insect rearing tent to maintain whitefly-free conditions. Cotyledons were harvested 10 days after planting, and true leaves were collected 30 days after planting. Stem and root samples were also collected from the same plants. Twenty-five sampled tissues for each organ were bulked into five pools (five sampled tissues for one pool), and each pool was used for DNA extraction with the Viral Gene-spin^TM^ Viral DNA/RNA Extraction Kit (iNtRON Biotechnology). DNA was isolated from each cotyledon and true leaf individually. PCR was performed as described above. The detection rate range for seeds and the detection rate for embryos varied from the minimum and maximum numbers because the infection rates were calculated based on the number of bulks that were confirmed to be TYLCV infected by PCR.

### Identification of the TYLCV-tolerant loci using sequence-characterized amplified region/cleaved amplified polymorphic sequence (SCAR/CAPS) markers

To identify tolerant loci (*Ty*-1, 2, and 3) in the TYLCV-tolerant Bacchus cultivar tomato, sequence-characterized amplified region/cleaved amplified polymorphic sequence (SCAR/CAPS) markers of *Ty*-1, *Ty*-2 and *Ty*-3 introduced in previous studies^39^,^40^,^41^ were detected. PCR was performed with specific primers (Table 3). PCR products for the *Ty*-2 and *Ty*-3 markers were directly loaded on an agarose gel, and amplicons for the *Ty*-1 marker were eluted and digested with *Taq*I restriction enzyme at 65 °C for 1 hr. *Taq*I-treated products were also confirmed through agarose gel electrophoresis.

### Real-time quantitative PCR

To analyze the relationship between TYLCV DNA amounts and TYLCV susceptibility, amounts of genomic DNA of TYLCV were tested by real-time quantitative PCR from the leaves of four-week-old seed-borne TYLCV-infected plants of two different cultivars, TYLCV-susceptible cv. Seogwang and TYLCV-tolerant cv. Bacchus. Reactions were performed using the SYBR premix Ex *Taq* (*Tli* RNase H Plus, TaKaRa, Shiga, Japan) with specific primer sets (Table. 1). Cycling of PCR consisted of pre-denaturation at 95 °C for 5 min followed by 40 cycles of a denaturation step at 95 °C for 10 min, an annealing step at 60 °C for 15 sec, and an extension step at 72 °C for 20 sec using a Rotor Gene Q thermocycler (QIAGEN, Hilden, Germany). The annealing temperature was modified following the melting temperature of each primer, and each reaction was repeated at least three times. Relative TYLCV DNA amounts were normalized against T_1_ tomato. Data analyses were conducted by the 2^−ΔΔCt^ method^42^.

### Transmissibility test of seed-borne TYLCV to healthy tomato by whitefly

The transmissibility of seed-borne TYLCV in tomato to other healthy tomato plants was analyzed using sweet potato whiteflies. Thirty non-viruliferous *Bemisia tabaci* (Q biotype) were reared with one TYLCV-donor plant (TYLCV-infected tomato plants germinated from TYLCV-infected seeds, Seogwang cv.) and three TYLCV-receiver plants (TYLCV-free tomato plants, Seogwang cv.) in an insect rearing tent together at 25 °C with a day length of 12 hrs. Another insect tent rearing non-viruliferous whiteflies with healthy plants of tomato cv. Seogwang were prepared as a negative control group. After 8 weeks, each plant and whiteflies were harvested and analyzed for TYLCV infection by PCR, Southern hybridization and sequence analysis. This experiment was performed three times independently.

### Southern blot hybridization analysis

Southern hybridization analysis was conducted to confirm the viral replication of TYLCV in tomato plants using the modified method from Southern *et al.*^43^. Total DNA isolated from tomato tissues (15 μg) was loaded on 1% agarose gel. After the depurination, denaturation, and neutralization steps, DNA loaded on the gel was transferred to a positively charged nylon membrane (Hybond-*N*^*+*^ membrane, GE Healthcare Life Sciences, Waukesha, WI, USA) using the capillary transfer method for up to 16 h, and the transferred DNA was linked covalently to the nylon membrane using an ultraviolet crosslinker (UVC 500 crosslinker, GE Healthcare Life Sciences). The TYLCV DNA fragment, amplified with the detection primer set (Table 2), was gel purified and labeled with [α-^32^P] dCTP using the Rediprime II Random Primer Labeling System (GE Healthcare Life Sciences). Hybridization was conducted at 65 °C for 16 h. After washing, the membrane was then exposed to X-ray film (Kodak, Rochester, NY, USA) for approximately 48 h in a −70 °C freezer.

### Sequence analysis

PCR was performed in order to amplify the full-length DNA genome of TYLCV (Table 2). PCR products were ligated into a pGEM-T easy vector (Promega, Madison, WI, USA) and sequenced (Macrogen, Seoul, Korea). The sequences were analyzed using a multiple alignment program MultAlin (http://multalin.toulouse.inra.fr/multalin/)^44^ and basic local alignment search tool (BLAST, http://blast.ncbi.nlm.nih.gov/Blast.cgi)^45^.

## Additional Information

**How to cite this article**: Kil, E.-J. *et al.* *Tomato yellow leaf curl virus* (TYLCV-IL): a seed-transmissible geminivirus in tomatoes. *Sci. Rep.* **6**, 19013; doi: 10.1038/srep19013 (2016).

## Figures and Tables

**Table 1:** Infection rates for TYLCV from reproductive organs and seeds of TYLCV-infected tomato plants, cotyledons and true leaves of their offspring.

Rate of infection	Petal*	Stamen*	Pistil*	Fruit	Seed*	Embryo and endosperm*	Cotyledon	True leaf
Whitefly-mediated inoculation	5/5	5/5	5/5	6/6	6/6	12/12	22/26	41/45
	(20 ∼ 100%)	(20 ∼ 100%)	(20 ∼ 100%)	(100%)	(20 ∼ 100%)	(20 ∼ 100%)	(84.62%)	(91.11%)
Agro-inoculation	NT	NT	NT	5/5	5/5	12/12	21/26	55/75
				(100%)	(20 ∼ 100%)	(20 ∼ 100%)	(80.77%)	(73.33%)

^*^These results are from bulked samples with different tissues from five tomato plants shown typical TYLCV symptoms.

**Table 2:** Primer sets used for amplification of TYLCV in this study.

Primer name	Sequence (5′ – 3′)	Target size
*Detection PCR*		
TYLCV-F	GATGGCCGCGCCTTTTCCTTTTATGTGG	390 bp
TYLCV-R	GCTGCTGTATGGGCTGTCGAAGTTCAG	
*Real-time PCR*		
TYLCV-C1-F	GCTCGTAGAGGGTGACGAA	164 bp
TYLCV-C1-R	CACAAAGTACGGGAAGCCCA	
GAPDH-F	GCCACTCAGAAGACCGTTGA	188 bp
GAPDH-R	AGGTCAACCACGGACACATC	
*Full length sequencing*		
TYLCV-FL-F	CTTACTTATACCTGGACACCTAATGGC	2774 bp
TYLCV-FL-R	ACACCGATACACCGATTGCCATAG	

**Table 3:** Sequence-characterized amplified region/cleaved amplified polymorphic sequence (SCAR/CAPS) markers linked to the *Ty*-1, *Ty*-2 and *Ty*-3 locus on tomato chromosomes.

Target locus		Sequence (5′ – 3′)	Reference
*Ty*-1 (CAPS)	Forward	ATGAAGACAAAAACTGCTTC	Ji, *et al.*^41^
	Reverse	TCAGGGTTTCACTTCTATGAAT	
*Ty*-2 (SCAR)	Forward	TGGCTCATCCTGAAGCTGATAGCGC	Garcia, *et al.*^39^
	Reverse	AGTGTACATCCTTGCCATTGACT	
*Ty*-3 (SCAR)	Forward	GGTAGTGGAAATGATGCTGCTC	Ji, *et al.*^40^
	Reverse	GCTCTGCCTATTGTCCCATATATAACC	
